# Conceptualizing healthy cognitive aging: the role of time and variability

**DOI:** 10.3389/fnhum.2023.1240630

**Published:** 2023-09-13

**Authors:** Emma A. Rodrigues, Sylvain Moreno

**Affiliations:** ^1^School of Interactive Arts and Technology, Simon Fraser University, Surrey, BC, Canada; ^2^Circle Innovation, Vancouver, BC, Canada

**Keywords:** healthy cognitive aging, cognitive trajectories, individual profiles, subtyping, conceptualization

## Abstract

The interest in healthy cognitive aging (HCA) has increased substantially over the past decade. Researchers are interested in exploring how health can be promoted and cognitive decline mitigated when pathology is not present. Identifying the necessary strategies is crucial as the gradual accumulation of small declines can lead to negative effects on quality of life over time. However, the conceptualization of HCA is not agreed upon. In fact, authors often turn to the use of traditional pathology screeners in the context of HCA because of their clear threshold results and their wide use in the different fields. This leads to the assumption that individuals are either cognitively unhealthy and therefore may have some form of dementia or are dementia-free and cognitively healthy. We believe that this view is an overly simplistic approach to the understanding of the aging process. In this work, we explore how HCA has been defined and conceptualized within the different fields. We further discuss how time and variability are key concepts that are often missing when studying HCA and propose a definition that aims to unify the findings from the multidisciplinary research that studies HCA and simplify the translation of knowledge. Incorporating these two novel dimensions to the study of HCA has already been proposed methodologically but has yet been discussed at the conceptual level. We believe that the proposed new approach will allow the identification of individual factors that cause changes in cognitive health and will help build new cognitive health strategies and mitigate further declines.

## Introduction

Research on Healthy Cognitive Aging (HCA) has increased 7-fold in the past decade (Krivanek et al., 2021). Reasons for this increase may be tied to many researchers challenging the assumption that dementia is an inevitable consequence of aging (Qiu and Fratiglioni, 2018). In fact, studies have suggested that the cognitive process associated to HCA presents smaller variations compared to pathology, but that the gradual accumulation of these declines could have negative effects on quality of life. Therefore, identifying when these changes occur is crucial as it can lead to the mitigation or reversion of small cognitive declines (Salthouse, 2019). However, current standard assessment tools rely on pathology screeners, which may not be sufficiently sensitive to detect these subtle changes (Oschwald et al., 2020).

In fact, authors often turn to the use of traditional pathology screeners in the context of HCA because of their clear threshold results and their wide use in the field (Roalf et al., 2013). However, in doing so authors conclude a false assumption, which is considering non-pathological individuals as cognitively healthy (Sánchez-Izquierdo and Fernández-Ballesteros, 2021). The implications of this false assumption are twofold. First, it assumes homogeneity of the population as the healthy aging population is all treated similarly (Kaneko et al., 2021). Given that the goal of the selected tests is to identify pathologies, meaningful differences in cognition within healthy older adults may not be detected. This will drive the assumption that none or only insignificant changes have occurred, which leads to the second assumption, as it assumes that potentially significant changes in cognitive health are negligible, as tests are not sufficiently demanding to avoid ceiling effects (Rabbitt, 2002). Subjective memory complaints (SMC) are an example of how the clear thresholds of pathology screeners may hamper the detection of meaningful changes in older adults. These complaints refer to those made by individuals with cognitive complaints but no clear impairment on objective psychometric testing, however, these appear to be associated with faster progression to dementia (Jessen et al., 2010; Steinberg et al., 2013). Nonetheless, individuals with SMC are considered to be within normal functioning, and major recommendations may only be provided once the cognitive loss has reached quantitative relevance through the pathology screener (Steinberg et al., 2013). Despite the abundant use of these methodological approaches, several authors agree that considering individuals as either neuropathological and therefore, cognitively unhealthy, or pathology-free and cognitively healthy, has become an overly simplistic view of the aging process (Blazer et al., 2015a).

We believe that at the core of this problem lies an unclear definition of HCA. Given the multidisciplinary nature of the field of HCA (Hofer and Alwin, 2008), it is important to establish a definition that facilitates research towards a common goal. Further, Liverman, Yaffe & Blazer highlight that one of the core motivations to develop operational definitions within cognitive aging is to allow comparisons across studies (Liverman et al., 2015). When the motivation for studying HCA is to promote good cognitive health across the lifespan, multiple lenses are needed, as the cognitive aging process is the result of a conjunction of factors that shape performance over time (Hofer and Alwin, 2008). To evaluate this process, attention needs to be given to a range of sub-disciplines within HCA such as those that focus on neuronal structure and function, behavior and social factors which may contribute to dynamic adaptative processes of cognitive health (National Research Council, 2000). When undefined terms are employed across disciplinary boundaries or translated across fields, confusion, misunderstanding, and misinterpretation can occur (Cherbuin et al., 2021). Furthermore, inconsistent definitions can limit the interpretability of results and delay the potential for interdisciplinary research progression (Quinn et al., 2020). This is of important consideration given that despite the important advances being achieved in each of the different disciplines, without a common language, knowledge cannot be easily translated.

This commentary paper aims to discuss why HCA cannot be examined through the lens of pathological aging alone and how an unclear definition and new findings, such as the importance of subtyping, are at the core of the need to redefine HCA. Different authors have proposed definitions surrounding HCA, but to date, no consensus has been achieved. In this work we will explore some of the different proposed definitions, highlight their limitations and how they may overlap.

To address this goal, we explored the literature surrounding HCA. To the best of our knowledge, this is the first paper to compare and analyze a range of proposed definitions of HCA in different scientific fields. Most of the available literature that has proposed conceptual definitions, does so within the framework of their scientific field, limiting is translation across disciplines. In exploring the literature, we found that the term “healthy cognitive aging” is typically assumed to be the opposite of neuropathological aging (Snowdon, 2003; Bartrés-Faz and Arenaza-Urquijo, 2011; Gallagher et al., 2019), and that concepts such as normal and successful cognitive aging are often included in this term. Successful cognitive aging in particular has been vastly discussed, mainly within the field of gerontology, however, despite aiming to represent the highest functioning older adults, the majority of the proposed definitions were still mainly based on the absence of disability (Depp and Jeste, 2006). Therefore, when searching the different proposed definitions available in the literature, we included not only “healthy cognitive aging” but also “normal” (referring to cognitive aging compared to a norm) and “successful cognitive aging” (referring to cognitive aging at a high functioning level into late years in life) (NCA and SCA, respectively) as search terms.

The need for a definition on HCA often appears to be motivated by the increasing demand of better understanding available data. This data is commonly census data, behavioral data or direct measures of the brain (Blazer et al., 2015b). As a result, the scientific fields included in this work were selected based on the extensive literature available in assessing these types of data. We identified that these different data formats were mainly assessed by three core fields – Neuroscience, Psychology and Gerontology. Despite the sometimes-overlapping nature of the selected works, they were categorized based on the journal they were published in and the departmental affiliation of the authors. Table 1 highlights some of the different definitions of HCA, NCA and SCA, and the focus of the proposed definition. Section 1.1 of Table 1 highlights the work from the Neuroscience field. Section 1.2 of Table 1 includes the work from the Psychology field. And Section 1.3 of Table 1 highlights work from the Gerontology field.

**Table 1:** List of HCA definitions within the neuroscience, psychology, and gerontology fields.

Reference	Proposed definition	Focus	Term defined
1.1 Neuroscience			
Hendrie et al. (2006)	“Healthy cognitive aging is not just the absence of cognitive impairment, but the development and preservation of the multi-dimensional cognitive structures that allow the older adult to maintain social connectedness, and ongoing sense of purpose, and the abilities to function independently, to permit functional recovery from illness and injury, and to cope with residual cognitive deficits.”	Link to behavioral preservation Distinction from pathology Integration of multiple dimensions	Healthy Cognitive Aging
Daffner (2010)	“An alternative definition of normal cognitive aging suggests that it represents “non-pathological” aging, that is, older individuals without identifiable diseases or conditions (e.g., Alzheimer’s disease (AD), cerebral vascular disease) that negatively impact the central nervous system. However, many would argue that there is a continuum between normal and pathological cognitive aging.”	Distinction from binary view highlighting the possibility of a continuum Link to behavioral consideration Non-pathological aging	Normal Cognitive Aging
Greenwood et al. (2011)	“Successful cognitive aging (refers to) maintaining intact cognitive functioning while living to late old age”	Distinction between cognitive age and chronological age Maintenance of cognitive capacities	Successful Cognitive Aging
Malaspina et al. (2011)	“Successful cognitive aging broadly refers to the multi-determined process of preserving cognitive abilities or exhibiting less-than-expected decline in neural structure and function typically associated with aging and its comorbidities.”	Integration of multiple dimensions Includes functional and structural changes Maintenance of cognitive capacities	Successful Cognitive Aging
Depp et al. (2012)	“An additional approach to operationalization would be to define success in reference to preservation of past performance—i.e., maintaining levels of cognitive performance attained at mid-life.”	Consideration of individual changes Maintenance of cognitive capacities	Successful Cognitive Aging
Dumas (2015, 2017)	“Normal aging has been defined as aging changes that occur in individuals free of overt diseases of the nervous system. The goal of successful aging is to maintain intact cognitive functioning all the way until death. Normal cognitive aging is not dementia and does not result in the loss of neurons, rather, there are changes in brain functioning”	Maintenance of cognitive capacities Non-pathological aging Includes functional changes	Normal Cognitive Aging
Wallace et al. (2017)	“Successful cognitive aging was defined as the absence of neurocognitive impairment (as defined by deficits in tasks of episodic learning, information processing speed, executive function, and motor skills) depression, and functional impairment (instrumental activities of daily living).	Integration of multiple dimensions Includes cognitive and behavioral changes Non-pathological aging	Successful Cognitive Aging
Moore et al. (2018)	“Successful cognitive aging criteria (defined as the absence of objective neurocognitive deficits and subjective cognitive symptoms)”	Includes cognitive and behavioral changes Non-pathological aging	Successful Cognitive Aging
Stern et al. (2022)	“The use of a common vocabulary and operational definitions“(“… such as reserve and resilience…”) “will facilitate even greater progress in understanding the factors that are associated with successful aging”	Consideration of individual changes Maintenance of cognitive capacities Non-pathological aging Includes behavioral changes	Successful Cognitive Aging
1.2 Psychology			
Harrison et al. (2012)	“SuperAgers were defined as individuals over age 80 with episodic memory performance at least as good as normative values for 50- to 65-year-olds.”	Suggests a comparison to Normative scores Focus on a specific cognitive function Emphasizes importance of chronological age	Successful Cognitive Aging
Smith (2016)	“Aging with normal cognitive changes is defined by the subtle loss of cognitive and functional performance that occurs in normal aging, even when known brain diseases are absent”	Non-pathological aging Focus on cognitive and functional performance	Normal Cognitive Aging
Silverman and Schmeidler (2018)	“Successful cognitive aging is intact cognition in the oldest-old; we define resistant successful cognitive aging as successful cognitive aging despite high risk.”	Focus on intact cognition Suggests comparison of cognitive age to chronological age	Successful Cognitive Aging
Cabeza et al. (2018)	“Optimal aging defined as the situation in which cognitive abilities are preserved throughout aging. Healthy aging humans (defined here as aging in individuals who are apparently free of brain disease)”	Maintenance of cognitive functioning Non-pathological aging	Normal Cognitive Aging
Leal and Yassa (2019)	“Normal is “conforming to a standard; usual, typical, or expected.” Note that characterizations of normal versus abnormal in terms of pathology or illness are not the purpose of this chapter. In fact, the chapter does not attempt to characterize normal as a condition that is free of pathology (it is not) but merely as the typical or majority condition.”	Suggests a comparison to normative scores Highlights that NCA may not be the opposite of pathological aging	Normal Cognitive Aging
Oschwald et al. (2020)	“we refer to (healthy cognitive aging as the) processes that occurs in the absence of pathological cognitive impairments, as previous literature has not yet reached a consensus on the definition of healthy cognitive aging.”	Non-pathological aging	Healthy Cognitive Aging
1.3 Gerontology			
Massaldjieva (2018)	“Normal Cognitive Aging: Cognitive aging allowing for active and independent living Successful Cognitive Aging: Maintenance of intact cognitive functioning all the way until death”	SCA is linked to social preservation NCA is linked to maintenance of cognitive functions	Normal Cognitive Aging Successful Cognitive Aging
Mendoza-Ruvalcaba et al. (2018)	“The concept of cognitive functioning in normal aging has been defined as the functioning of the cognitive system, either in adaptation or alteration, which can generate a regression or successful management of the functions of daily life in older adults”	Focus on functional and social performance Maintenance of cognitive capacities	Normal Cognitive Aging
Cadar (2018)	“There is no clear consensus in defining healthy or successful cognitive aging, but it can be described as the maintenance of most cognitive abilities as until older age and a minimum variation in the spectrum of normal cognitive decline with aging.”	Maintenance of cognitive capacities Focus on cognitive performance Suggests a comparison to normative scores	Healthy Cognitive Aging Successful Cognitive Aging
Rowe and Kahn (1987)	“In many data sets that show substantial average decline with age, one can find older persons with minimal physiologic loss, or none at all, when compared to the average of their younger counterparts. These people might be viewed as having aged successfully with regard to the particular variable under study (…). They, in combination with people who show the typical nonpathological age-linked losses that we propose to designate usual, constitute the heterogeneous category of the normal (that is, nondiseased) in any age group.”	SCA linked to minimal physiologic loss NCA linked to non-pathological aging	Successful Cognitive Aging Normal Cognitive Aging
Clouston et al. (2020)	“Normal Cognitive aging is generally characterized by a slow but steady decay in fluid cognition (Salthouse, 2019) resulting from a seemingly unavoidable biological process (Richards and Deary, 2014).”	Focus on fluid cognition Steady decline of cognitive capacities	Normal Cognitive Aging

In Section 1.1, we observe that several of the proposed definitions discuss that a key determinant of HCA is the maintenance of cognitive health in older ages. We also observe the overlap between definitions of Normal, Healthy and Successful Cognitive Aging. An example of this is present in the definitions of HCA proposed by Hendrie et al. (2006), and SCA proposed by Wallace et al. (2017). Despite referring to seemingly different concepts, their definitions are very similar as they both discuss multiple levels to cognitive preservation. Another example of this overlap is found when the definitions describe the maintenance of cognitive health for all three different terms. This indicates that the differences between them are unclear and disputable. We also observe that several of the definitions frame HCA around the absence of pathology. Further, many describe that the goal of HCA is the preservation of this non-pathological state in different lenses of cognitive health – such as structural, functional, behavioral and social - which is a sub-optimal view, as previously discussed.

Section 1.2 highlights that included in some definitions of HCA are indicators of cognitive function such as memory (Harrison et al., 2012) or others (Smith, 2016; Cabeza et al., 2018). Moreover, some of the proposed definitions promote the use of normative scores as indicators of cognitive health (Harrison et al., 2012; Leal and Yassa, 2019), while others suggest that individual change is more informative (Cabeza et al., 2018). These distinct approaches are used when defining not only NCA but also SCA indicating once again that these terms may overlap in the literature but also that it is unclear how best to assess cognitive health in healthy aging. Finally, similarly to what was observed in Section 1.1, Section 1.2 also shows the binarized view that still dominates the literature viewing HCA as non-pathological.

Section 1.3 presents definitions identified within the field of Gerontology. Similarly, to the previous sections, here authors also suggest that HCA is defined in terms of a preservation or maintenance of cognitive health. Further, cognitive and functional preservation were also included in some of the definitions, similarly to what was presented in previous sections (Massaldjieva, 2018; Mendoza-Ruvalcaba et al., 2018). In defining HCA, a greater importance is given to social preservation which is reflected in independence and well-being. Finally, HCA and SCA were used interchangeably by Cadar (2018), emphasizing once again the overlap that the three terms have in the literature. Rowe and Kahn (1987) describe NCA as an umbrella term for usual cognitive aging and SCA. Lastly, the definitions provided in Section 1.3 are generalistic. In particular Rowe and Kahn (1987) and Clouston et al. (2020) refer to changes at the “physiological” or “biological” level rather than specifying behavior, structure or function, which creates ambiguity.

Overall, Table 1 provides possible definitions of HCA within different contexts. As highlighted individually before, the table identifies key issues with currently proposed definitions of HCA. Despite the unclear understanding of what HCA is and how it is distinguishable from NCA and SCA, the approaches to defining these terms vary across fields. In specific, in Section 1.1 we observe that the Neuroscience context includes a substrate focus and relies on quantifiable measures. Here the definitions include changes to neural structures and functions or variations in tasks related to specific cognitive functions. This is highlighted in the definition proposed by Malaspina et al. (2011), that discusses the “multi-determined process of preserving cognitive abilities or exhibiting less-than-expected decline in neural structure and function typically associated with aging and its comorbidities.” In this example, the authors discuss HCA in the context of brain structure and function, in comparison to pathological aging. Here, authors assume a homogeneity of the healthy population as they are highlighting that all individuals who do not present significant pathological declines are aging successfully. This description is observed across most of the proposed definitions within the Neuroscience field [eg.: Daffner (2010), Dumas (2015), Wallace et al. (2017), and Moore et al. (2018)]. However, homogenizing the population in such a manner neglects that smaller reversible cognitive changes may be captured to prevent greater ones (Salthouse, 2019). Further it neglects the various dynamics that may occur within HCA leading to faster or slower changes in cognitive health (Wu et al., 2020). In Section 1.2, we observe that within the Psychology context, greater emphasis is given to behavioral performance. We also observe that unlike what is observed in Section 1.1, here the authors use terms that provide a qualitative assessment of cognitive health such as “subtle loss” or “at least as good as,” which may lead to subjective interpretations. As an example, Smith discusses that “aging with normal cognitive changes is defined by the subtle loss of cognitive and functional performance that occurs in normal aging, even when known brain diseases are absent.” In this example, in addition to the qualitative assessment of cognitive health, the authors emphasize a binary view on the aging process (healthy brain vs. diseased brain). These limitations are present in other proposed definitions within the Psychology field [eg.: Cabeza et al. (2018), Silverman and Schmeidler (2018), and Oschwald et al. (2020)]. Lastly, Section 1.3 describes literature from the Gerontology context and highlights that the focus of these proposed definitions surrounds social preservation. As an example Massaldjieva (2018) discusses that “Normal Cognitive Aging (reflects) Cognitive aging allowing for active and independent living; Successful Cognitive Aging (reflects) Maintenance of intact cognitive functioning all the way until death.” The clear distinction between both these terms is done in the context of behavioral outcomes in which individuals maintain their independence and well-being. This is also observed in other proposed definitions within the Gerontology field [eg.: Rowe and Kahn (1987) and Mendoza-Ruvalcaba et al. (2018)]. Overall, each of the assessed fields highlights important factors that accompany the cognitive aging process, be-it at the neurological (eg.: structure and function), the performance (eg.: reaction times and words recalled) or the behavioral (eg.: independence and well-being) level. These aspects of the aging process are undoubtedly important to further understand what occurs throughout this process, however, the specificity of the proposed definitions limits its applicability outside of the current field neglecting the translation of findings across disciplines.

In investigating the different proposed definitions, we created 5 categories to facilitate their comparison within and across fields. Table 2 presents the comparisons of the definitions presented above across these categories. The first category is “No Pathology,” in which the absence of pathology was considered an indicator of HCA. The second category is “Behavioral Preservation” in which different cognitive assessments indicate a level of cognitive function. This category represents the accessible pencil and paper tests, which are often cost and time effective. The third and fourth categories (“Structural” and “Functional Preservation,” respectively) are reflective of neuroimaging assessments which provide quantitative measures of brain health. These approaches are less accessible, costly and time consuming. The final category is “Social Preservation” in which independence and well-being are considered indicators of HCA.

**Table 2:** Comparison of definitions across 5 categories: “No Pathology”; “Behavioral Preservation”; “Structural Preservation”; “Functional Preservation”; “Social Preservation.”

	Reference	No Pathology	Behavioral Preservation	Structural Preservation	Functional Preservation	Social Preservation
Neuroscience	Hendrie et al. (2006)	x	x			x
	Daffner (2010)	x				x
	Greenwood et al. (2011)		x			
	Malaspina et al. (2011)		x	x	x	
	Depp et al. (2012)		x			
	Dumas (2015)	x	x			
	Wallace et al. (2017)	x				x
	Moore et al. (2018)	x				
	Stern et al. (2022)	x	x	x	x	
Psychology	Harrison et al. (2012)		x			
	Smith (2016)	x	x		x	
	Silverman and Schmeidler (2018)		x			
	Cabeza et al. (2018)		x			
	Leal and Yassa (2019)	x				
	Oschwald et al. (2020)	x				
Gerontology	Massaldjieva (2018)		x			x
	Mendoza-Ruvalcaba et al. (2018)		x			x
	Cadar (2018)		x			
	Rowe and Kahn (1987)	x	x			
	Clouston et al. (2020)		x			

## In reviewing the different proposed definitions in the literature, we observe three main limitations

### Many current definitions are framed around pathology

As observed in Table 2, many of the proposed definitions of HCA suggested that it may be the opposite of pathology. This approach promotes a binary perspective on aging, suggesting that if an individual is free of neurological impairment, then the individual is considered to be healthy. A limitation linked to this perspective is tied to the methodological approaches often used in research settings, such as the Mini Mental State Examination (Roalf et al., 2013).

Let us consider the binary view as true for the following argument: if health is the opposite of pathology, pathology screeners can be used to assess whether an individual is healthy (as they would not score below the pathology threshold to be diagnosed). However, pathology screeners have been designed to have high specificity and sensitivity to the pathology they were created to detect. Therefore, an individual with some degree of pathology may be deemed healthy only because the disease has not sufficiently progressed. Given the healthy prognosis, no further investigation is typically carried out.

Overall, if a comparison to a pathological state is not included in the definition of HCA, pathology-specific testing would no longer be an appropriate approach. Instead, researchers would have to identify alternative methods to measure health in cognitive aging that is sensitive to the subtle changes associated with the aging process. An example of the importance of this tool is made clear when SMC are discussed. SMC are not detected through the use of pathology tests but are a key indicator of accelerated cognitive decline and are linked to increased risk of dementia (Steinberg et al., 2013). As described by Altman and Bland (1995), “absence of evidence is not evidence of absence.”

### Current definitions are discipline specific

The tables above also highlight that in some cases, the applicability of the definitions proposed are only suitable for a specific field. As an example, the definition proposed by Harrison et al. (2012), may not be adequate for researchers interested in assessing HCA in individuals under the age of 80 or beyond the scope of episodic memory performance. Similarly, the definition proposed by Massaldjieva (2018).

may not be suitable for researchers interested in studying brain and cognitive reserve in HCA. The National Research Committee on Assessing Behavioral and Social Science on Aging discussed that when projects are highly conservative in their research disciplines, they fail to convert findings into useful and usable applications (National Research Council, 2007; Hofer and Alwin, 2008).

Overall, when definitions are discipline specific, they limit the transferability of the different concepts across fields. Discipline specific definitions also carry increased assumptions associated to that given field. This is a crucial point of consideration given that a consensus would allow researchers to work towards a common goal. Given the interdisciplinarity of HCA and the many lenses that have been used to study it, a global definition would facilitate the transfer of knowledge across fields, which is currently challenging.

### The proposed definitions do not consider individual variability over time

One dimension absent from the multiple proposed definitions of HCA is the “non-stationary” aspect of health. Indeed, the definitions above highlight that few authors consider differences in individual variability between persons or over time. A reason for this may be linked to (once again) the pathological context that HCA is often framed around. The changes in HCA will be much smaller than those observed in pathological aging which can lead to them being neglected. However, the gradual changes that accumulate over time can result in negative effects on quality of life, and should therefore be considered (Salthouse, 2019).

Within the context of HCA, irrespective of absolute scores in cognitive function at a given point in time, longitudinal measures of cognitive change can capture meaningful variation in declines that are relevant for adults’ everyday lives (Tucker-Drob, 2019). As these changes may vary between and within individuals, assessing the rate of cognitive change can provide a better understanding of person-specific strategies that need to take place to mitigate declines.

Furthermore, this limitation is peculiar, as there is a big body of literature supported by both cross-sectional and longitudinal studies, that indicates that older adults show a great range of variability in cognitive functioning (Sánchez-Izquierdo and Fernández-Ballesteros, 2021). In specific, different theories have been proposed to explain why some individuals may present differences in neuronal and cognitive resources but appear behaviorally healthy [e.g.: brain reserve (Katzman et al., 1988), cognitive reserve (Stern, 2002), compensation (Cabeza et al., 2002), scaffolding theory of aging and cognition (Park and Reuter-Lorenz, 2009), maintenance (Nyberg et al., 2012) and selection-optimization-compensation model (Baltes and Baltes, 1990)]. A relevant review of the literature authored by Nyberg & Pudas propose a model that highlights the possible drivers of variability as well as the many bi-directional interactions that occur throughout the aging process. In this work, the authors discuss the role of genetics, lifestyle influences and individual changes in brain function and integrity (either associated to age-related changes or strategic maintenance and compensation processes). Here, the authors focus on “successful memory aging” and “usual memory aging” as a strategy to reduce the complexity of the umbrella term that cognitive aging can encapsulate. A key feature of the proposed model is the idea that there are several paths that lead to HCA and that HCA can differ in its expression (Nyberg and Pudas, 2019).

Given the relevance of the heterogeneity present in cognitive aging, apparent through extensive reviews such as the one discussed above by Nyberg and Pudas (2019), the field has focused on understanding it and exploring its drivers. To that end, some authors have proposed subtyping as a suitable conceptual approach which defines categories of participants based on their genotypic and/or phenotypic features (Moreno et al., 2023). Some authors discuss subtyping in the context of structural brain atrophy, as the mechanisms surrounding differences in atrophy rates may differ (Gorbach et al., 2017; Nyberg and Pudas, 2019; Nyberg et al., 2022). Other authors have discussed subtyping in the context of cognitive trajectories, investigating the drivers of differences in trajectories (Wu et al., 2020). Subtyping has also been described in the context of patterns of cognitive dispersion, which refers to the variability observed in individual performance across tasks (Hilborn et al., 2009) and in the context of SMC which may be associated with different personality traits leading to the need of subtype specific recommendations (Steinberg et al., 2013). Lastly, we have recently discussed possible population differences in environmental susceptibility within the cognitive aging context and have highlighted that some individuals are highly sensitive to their environmental surroundings while others are not (Rodrigues et al., 2022). Overall, understanding the heterogeneity in cognitive aging has been a growing research avenue, and its relevance in describing the HCA population has been significantly acknowledged. However, these concepts and related findings have yet been included in proposed definitions of HCA.

Given the previously presented work and the gaps highlighted by Table 2, we infer that the way in which the healthy aging population has been defined is sub-optimal. As Lindenberger (2014) and Salthouse (2019) have shown, and the present paper has highlighted, it is crucial that we incorporate two key dimensions in the conceptualisation of HCA: variability and time.

Variability: To detect the smaller changes in cognitive health linked to the highly variable population of healthy aging, different methodological techniques have been proposed. These approaches range from a characterization of a normative trajectory of HCA as proposed by Salthouse (2019) and Tucker-Drob (2019), to the assessment of cognitive scores over time at an individual level, as proposed by Mella et al. (2018). Further, some authors have also explored the possible role of individual daily changes in cognition in the early detection of subtle cognitive loss in HCA [eg.: Hilborn et al. (2009) and Verhagen et al. (2019)]. As an example, Hultsch et al., discuss that intraindividual variability may be an indicator of processing efficiency, providing information on cognitive functioning but also an agent of developmental change responsible for a range of age-related cognitive transformations (Hultsch et al., 2011).

Despite the methodological advances and the valuable distinction between healthy and pathological cognitive health, a limitation of the former approach lies with averaging of different scores which may fail to capture important idiosyncrasies relevant in alternative trajectories. On the other hand, the latter approach can lead to challenges in obtaining a reliable and noise-free assessment of intraindividual variability. As an alternative approach that addresses the limitations of the normalization or individualization strategies previously described, Rodrigues et al. (2022) and others (McCarrey et al., 2016; Gorbach et al., 2017; Wu et al., 2020; Nyberg et al., 2022) propose a profiling/subtyping approach through which smaller changes can be captured but noise can be better dealt with. Subtyping refers to the categorization of groups of individuals according to genotype and/or phenotype features (Moreno et al., 2023).

Time: Regarding the time component associated with HCA, researchers suggest that not all individuals age the same, and that while some individuals experience little change, others experience complex non-linear dynamics (Wu et al., 2020; Laplume et al., 2022). Explanations for these differences are described in longitudinal studies that reveal that individual differences in cognitive performance increase from early to late adulthood to which both genetic and environmental factors contribute to Sánchez-Izquierdo and Fernández-Ballesteros (2021). Further, despite the overall decline trend and the between-person differences in cognitive health, individual heterogeneity increases from early to late adulthood, with individual cognitive trajectories being malleable (Lindenberger, 2014). The study of HCA through the use of cognitive trajectories, as is done by Salthouse (2019) and Tucker-Drob (2019), highlights the temporal importance associated with the older adult population. This perspective implies a longitudinal approach to measure HCA.

The implementation of these concepts has been facilitated in the past years due to the increasing availability of health data. In fact, the number of publications on big data and health has grown exponentially in the last decade (Pastorino et al., 2019). Currently, different types of data can be collected over large periods of time, ranging from electronic healthcare records, genomic and pharmaceutical data, and clinical trials to mobile apps, sensors and information on well-being, behavior and socio-economic indicators. Biomedical research in specific, produces large amounts of data that researchers can access for secondary use through a variety of data repositories and biobanks (Ienca et al., 2018). Examples of companies that have focused on collecting and analyzing healthcare data include Microsoft Cloud,^1^ IBM Watson Health,^2^ OptumInsight^3^ and Google Health.^4^

## Conclusion

In this work we reviewed proposed definitions of HCA found in several fields of the literature. Through this process, we identified that cognitive health in healthy aging varies throughout the individual’s lifespan and is related to factors such as general health and social engagement (Wang et al., 2019). We identified that there is wide consensus that HCA is not the opposite of pathological aging (Irwin et al., 2018). Instead, HCA may be the result of modifiable and adaptable intrinsic processes that allow for an individual to maintain well-being, social connectedness, and independence in older age (Cabeza, 2004). Further, this work allowed us to explore how discipline specific approaches may be sub-optimal approaches to understand changes in HCA. Cognitive measures (mostly acquired through pencil and paper assessments) alone cannot predict individual declines. Similarly, brain measures (neuroimaging assessments) alone may fail to capture compensation mechanisms of individuals that display clinical levels of neural degeneration but function at healthy cognitive levels (those with higher reserves) (Bialystok et al., 2021). Lastly, we identified a gap in the literature in which while the conceptualization of HCA includes key characteristics of variability and time, its traditional measures of cognitive health in aging do not. We believe that the reason for this disconnect is tied to the unclear definition of HCA.

Through this paper, we have discussed that the core concepts of Variability and Time give rise to the notion of trajectory, which through repeated appropriate assessments allow for an increased likelihood of capturing change and a better understanding of HCA. From a methodological perspective, researchers have started exploring heterogeneity in the context of population subtyping. This approach requires a nonlinear understanding of cognitive aging and credits part of the heterogeneity to previously viewed as “noise,” as “subtype-specific.” We believe that it is crucial to integrate these principles in the conceptualization of HCA in a way that is meaningful across disciplines. Overall, given the current literature, the advantages and limitations of the different proposed definitions, and the key highlighted gaps, we propose that HCA is defined in the following way:

*Healthy Cognitive Aging is the cognitive trajectory characterized by the individual profile that mitigates the effects of age-related social, behavioral and neural decline.*

This definition provides a clear conceptualization of HCA while allowing for individual differences to be considered. It defines HCA in a longitudinal manner, not restricting its assessment to a specific discipline. It further considers age-related changes within the older adult population, recognizing that these may not be the same across the population. Finally, it is also flexible in how these age-related changes may be interpreted (either behaviorally, structurally, functionally or socially).

Incorporating these two novel dimensions to the study of HCA has already started (Lindenberger and Oertzen, 2006; Salthouse, 2019; Wu et al., 2020) and it is changing our scientific approaches. These approaches force scientists to rethink their methods and the way they conceptualize cognitive aging. The future of this field is the understanding of population subtyping and personalized trajectories. This new approach will allow the identification of individual factors that cause changes in cognitive health, help us build new cognitive health strategies and mitigate further declines.

## Data availability statement

The original contributions presented in the study are included in the article/supplementary material, further inquiries can be directed to the corresponding author.

## Author contributions

ER and SM provided substantial contributions to the conception of the work and contributed to drafting and revising the work critically for important intellectual content. All authors contributed to the article and approved the submitted version.

## Conflict of interest

The authors declare that the research was conducted in the absence of any commercial or financial relationships that could be construed as a potential conflict of interest.

## Publisher’s note

All claims expressed in this article are solely those of the authors and do not necessarily represent those of their affiliated organizations, or those of the publisher, the editors and the reviewers. Any product that may be evaluated in this article, or claim that may be made by its manufacturer, is not guaranteed or endorsed by the publisher.

## Footnotes

^1^https://www.microsoft.com

^2^https://www.ibm.com

^3^https://www.unitedhealthgroup.com

^4^https://health.google/
